# Extended-Release Immunotherapy-Eluting Embolics

**DOI:** 10.3390/cancers18162591

**Published:** 2026-08-12

**Authors:** Imran Shair Mohammad, Anup Kumar Patel, Steven T. Rosen, Marcin Kortylewski, Jonathan Kessler, Julie Ressler, Chandana Lall, Catalina Guerra, Jason Chiang, F. Edward Boas

**Affiliations:** 1 Department of Radiology, City of Hope Medical Center, Duarte, CA 91010, USA; imohammad@coh.org (I.S.M.);; 2 Department of Hematology & Hematopoietic Cell Transplantation, City of Hope Medical Center, Duarte, CA 91010, USA; 3 Department of Immuno-Oncology, City of Hope Medical Center, Duarte, CA 91010, USA; mkortylewski@coh.org; 4 Lundquist Institute, Torrance, CA 91010, USA; 5 Department of Radiological Sciences, University of California, Los Angeles, CA 90095, USA; cjchiang@mednet.ucla.edu

**Keywords:** immunoembolization, drug-eluting embolics, intratumoral immunotherapy, in situ cancer vaccination, liver metastases, Oncopig

## Abstract

**Purpose**: Systemic immunotherapy is less effective in patients with liver metastases. Immunoembolization could potentially overcome the immunosuppressive tumor microenvironment, but the optimal immunotherapy agent, embolic, and release kinetics are unknown. **Methods**: A wide range of immunotherapy agents (cytokines, small molecules, and oligonucleotides) were loaded onto various embolics (stabilized lipiodol emulsions, microspheres-in-lipiodol, LC beads, and PLGA microspheres). Drug loading and release kinetics were evaluated in vitro. A pilot study of transarterial immunoembolization was performed in a pig liver tumor model, using extended release lipiodol/CpG-STAT3ASO formulations. CpG-STAT3ASO is a first-in-class dual-function immunotherapy agent that both stimulates the anti-tumor immune response, and counters immunosuppression in immunologically cold tumors. Safety, pharmacokinetics and efficacy were evaluated in pigs. **Results**: Immunotherapy-loaded embolics released drug with a half-life ranging from days to weeks in vitro, and up to four days in pig liver tumors (depending on the formulation). A stabilized lipiodol emulsion demonstrated 100% burst release (within five minutes after immunoembolization), compared to <1% burst release with microspheres-in-lipiodol. Immunoembolization using lipiodol/CpG-STAT3ASO resulted in decreased size of pig liver tumors, compared to bland embolization. No adverse events were seen, based on clinical, laboratory, and radiographic evaluation, but peritoneal adhesions were observed in association with immunoembolization using microspheres-in-lipiodol. **Conclusions**: Extended-release immunotherapy-loaded embolics generate sustained intratumoral delivery of immune stimulants. A pilot study of immunoembolization of pig liver tumors showed decreased tumor size, compared to bland embolization, with no autoimmune adverse events.

## Simple Summary

Immunotherapy can help the immune system attack cancer cells, but it often works poorly for liver metastases, where immune responses are naturally suppressed. This study proposes a new way to deliver immune-stimulating drugs directly into liver tumors via the tumor’s blood supply, using drug carriers that slowly release the drug into the tumor over time (immunoembolization). We developed and tested several different drug carriers, including microspheres and emulsions, and showed that the immunotherapy drug is released into the tumor over days to weeks, resulting in liver tumor shrinkage in pigs. Extended-release immunoembolization has the potential to maximize immune activation in liver metastases (in situ vaccination) while reducing autoimmune side effects.

## 1. Introduction

Arterially directed therapies are typically performed using embolics loaded with radioisotopes (radioembolization) or cytotoxic chemotherapy (chemoembolization). A wide range of newer drugs are now available, which would benefit from locoregional delivery to increase efficacy and reduce extrahepatic side effects [1,2,3,4]. For example, immunoembolization [5,6,7,8] of liver tumors enables treatment of both intrahepatic and extrahepatic [6,8] disease, with low toxicity. Embolics loaded with anti-angiogenic agents [9,10,11,12], or hypoxia-activated prodrugs [13,14] have also shown promising results.

Chemoembolization or radioembolization of liver tumors can induce immunogenic cell death [15,16]. However, abscopal effects remain rare, even when combined with systemic checkpoint inhibitors [17,18]. Immune tolerance in the liver limits the effectiveness of checkpoint inhibitors in patients with liver metastases [19]. T cells recognize tumor antigens in liver tumors, but are actively suppressed [19,20]. Immunologically “cold” tumors (such as pancreatic cancer and microsatellite-stable colon cancer) have a poor response rate (<20%) to systemic immunotherapy. Immunoembolization could potentially overcome the immunosuppressive tumor microenvironment in the liver by delivering powerful immune stimulants into liver tumors, while avoiding normal tissue [5,6,7,8].

However, there is no general method for loading drugs onto embolics. For example, current drug-loadable microspheres (LC beads) are negatively charged and can only be loaded with positively charged drugs, such as doxorubicin. There are no FDA-approved microspheres that can be loaded with negatively charged drugs. Water-soluble drugs can be difficult to incorporate into PLGA microspheres [21]. Protein drugs can denature in water-in-oil emulsions [22].

**Figure 1:**
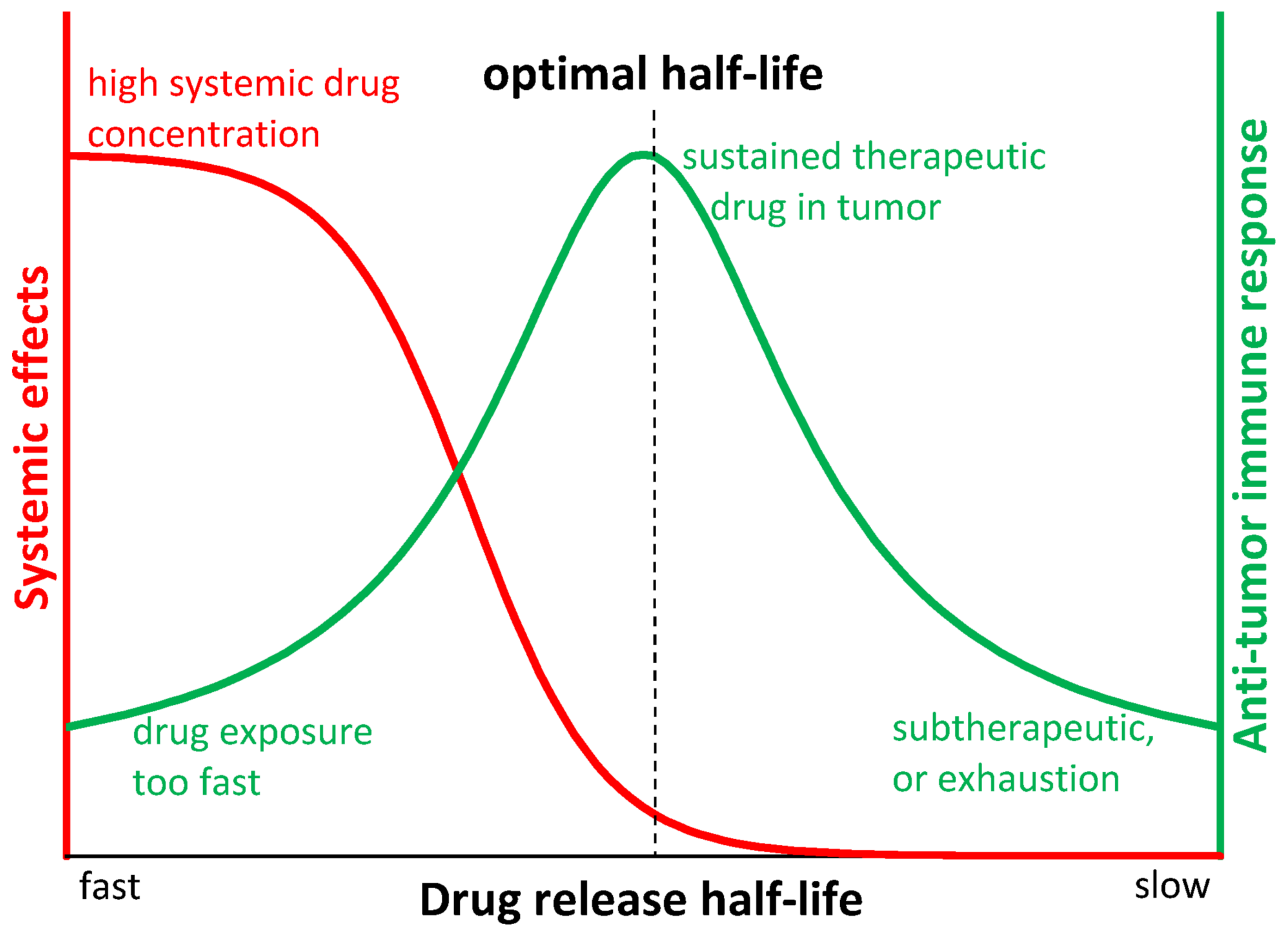
**Pharmacokinetics of locoregional immunotherapy (schematic diagram)**. Sustained local release of immunotherapy agents reduces systemic effects (red), and maximizes the anti-tumor immune response (green). However, if release is too slow, the intratumoral drug concentration could become subtherapeutic, or T cells could become exhausted.

Pharmacokinetics of drug release from drug-loaded embolics is a critical factor affecting safety and efficacy. For example, some drug-loaded embolics have high burst release [23], resulting in immediate systemic release of 45% of the drug, potentially causing systemic toxicity. The half-life of drug release is another key variable (Figure 1). Rapid drug release from an embolic results in decreased intratumoral exposure to the drug and a higher systemic drug concentration. Our limited control of drug loading and release kinetics remains a significant barrier to the development of effective immunotherapy-loaded embolics.

**Figure 2:**
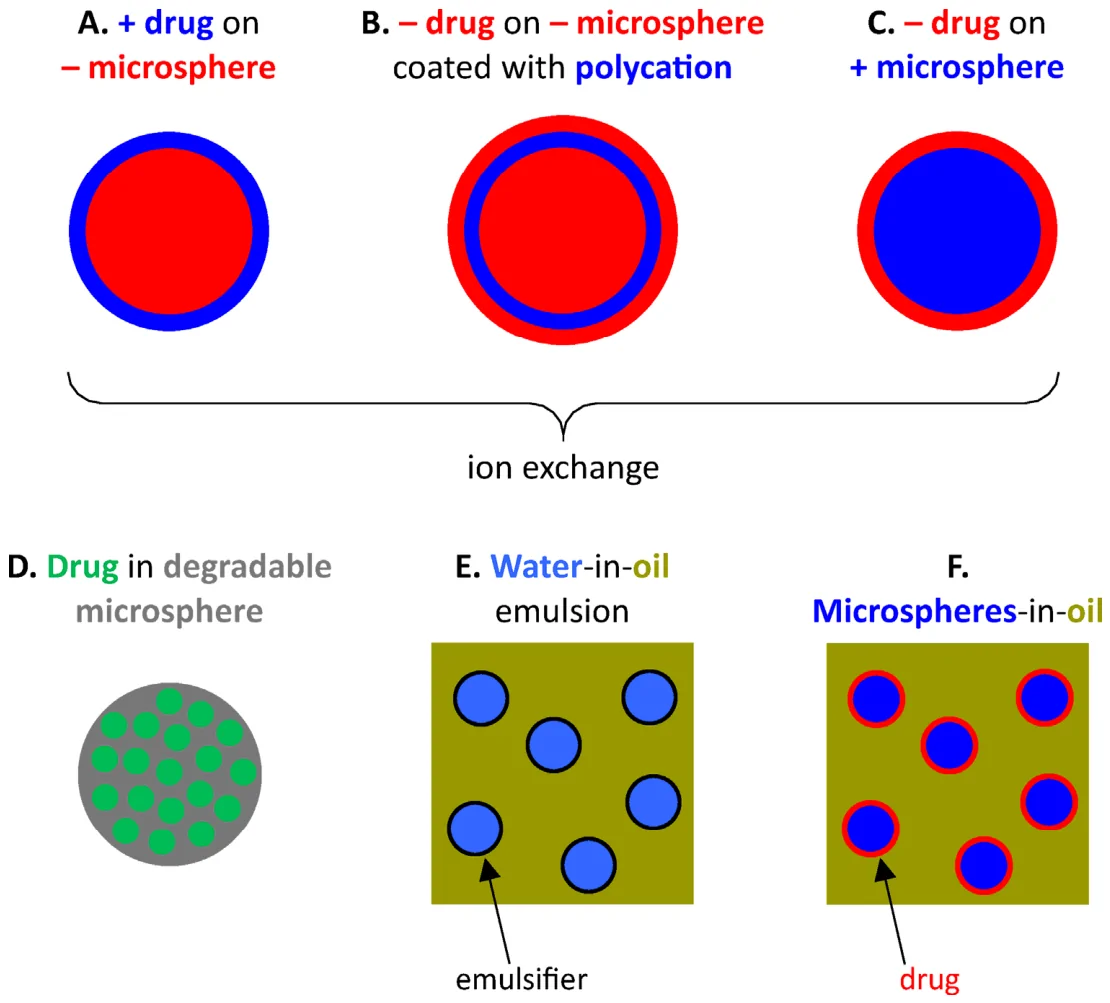
**General methods for making extended-release immunotherapy-eluting embolics**. (**A**). Negatively charged embolics (such as LC beads) can be loaded with positively charged drugs (ion exchange microspheres). (**B**). Negatively charged microspheres can be coated with positively charged polymers (such as protamine), then loaded with negatively charged drugs (layer-by-layer assembly). (**C**). Positively charged microspheres can be loaded with negatively charged drugs [4]. (**D**). Drugs can be incorporated into the matrix of degradable microspheres (such as PLGA). Hydrogel embolics can be loaded with water-soluble drugs, but drug release by diffusion is generally rapid. (**E**). A drug dissolved in water can be delivered in a water-in-oil emulsion, stabilized with emulsifiers. (**F**). Dehydrated drug-loaded microspheres can be suspended in oil.

Extended release immunoembolization has the potential to maximize immune activation in liver metastases (in situ vaccination), while reducing autoimmune adverse events. In this study, we develop new general methods for loading a wide range of immunotherapy agents onto various embolics (Figure 2), with a focus on FDA-approved embolics and excipients. We evaluate drug loading and release kinetics in vitro, and also perform a pilot study of safety, pharmacokinetics, and efficacy in a large animal liver tumor model.

## 2. Methods

### 2.1. Loading Cytokines and Positively Charged Small Molecules onto LC Beads (Ion Exchange)

LC beads (Boston Scientific, Marlborough, MA, USA) are negatively charged and can be loaded with positively charged drugs, as well as proteins containing both positive and negative charges. Loading is generally performed under low-salt conditions, at a pH where the microspheres and the drug are oppositely charged. Loading efficiency was calculated from the drug concentration in the supernatant, before and after loading (see Section 2.8).

*Loading imiquimod onto LC beads*: The imiquimod (5 mg) was dissolved in 5 mL of water, and the pH was adjusted to 4 by adding approximately 41 μL of 1 N HCl to protonate the amine, making it positively charged. Imiquimod solution was mixed with 2 mL of packed LC beads. Loading was performed for 2 h at room temperature, with periodic vortexing.

*Loading IL-2 onto LC beads*: IL-2 (0.1 μg in 1 mL PBS) was mixed with 0.1 mL of packed LC beads. Loading was performed for 2 h at 4 °C, on a rocker at 40 rpm.

*Loading IFN-γ onto LC beads*: IFN-γ (10 μg in 1 mL PBS) was mixed with 0.125 mL of packed LC beads. Loading was performed for 2 h at 4 °C, on a rocker at 40 rpm.

### 2.2. Loading Negatively Charged Drugs (Oligonucleotides) onto LC Beads (Layer-by-Layer)

Negatively charged LC beads can be coated with a positively charged polymer, such as protamine. The resulting positively charged beads can then be loaded with negatively charged drugs, such as oligonucleotides.

CpG is a single-stranded DNA that stimulates the innate immune response by activating TLR9. STAT3 is a master regulator of tumor immune evasion, and it can be blocked by an antisense oligonucleotide (ASO). CpG-STAT3ASO^Cy3^ is CpG, covalently linked to STAT3 ASO, and fluorescently labeled with Cy3. Porcine CpG-STAT3ASO^Cy3^ was synthesized in the City of Hope DNA/RNA synthesis core facility.

LC beads (0.09 mL) were coated with protamine (18 mg in 18 mL) in a glass vial at room temperature overnight, on an orbital shaker at 80 rpm, then washed 3 times with 500 μL of water. Zeta potential was measured to confirm a positive surface charge (Zetasizer Nano ZS, Malvern Panalytical, Malvern, UK). Protamine-coated LC beads were loaded with CpG-STAT3ASO^Cy3^ (0.5 mg in 1 mL) for 12 h in a glass vial at room temperature, on an orbital shaker at 20 rpm. Loading efficiency was calculated from the change in fluorescence of the supernatant, before and after loading (see Section 2.8).

### 2.3. Loading Drugs onto Quaternary Amine Microspheres (Ion Exchange)

Drugs were loaded onto quaternary amine microspheres (45–165 μm), as previously described [4]. Oligonucleotides, STING agonists, cytokines, and exosomes can be loaded onto quaternary amine microspheres.

### 2.4. Loading Drugs onto Degradable PLGA Microspheres

PLGA microspheres were prepared using an emulsification-solvent evaporation technique. A total of 300 mg of PLGA was dissolved in 3 mL of dichloromethane (DCM). The PLGA solution was added dropwise using an 18 G needle into 30 mL of 0.7% polyvinyl alcohol (70 kDa, partially hydrolyzed) in a beaker in an ice bath at 750 rpm. The emulsion was stirred at 550 rpm for 12 min in the ice bath, then at 450 rpm for 15 min at room temperature. A total of 20 mL of water was added, then the emulsion was stirred for 2.5 h. A sieve was used to remove microspheres larger than 300 μm. Microspheres were washed 3 times with water.

Hydrophobic drugs can easily be incorporated into PLGA microspheres, but water-soluble drugs are more difficult to incorporate [21]. Instead, we coat the PLGA microspheres with polydopamine (an adhesive polymer) or polyethyleneimine (a cationic polymer) to enable surface loading. Porous PLGA microspheres have more surface area for loading.

*Porous PLGA microspheres*. Porous PLGA microspheres were prepared using a double-emulsion solvent evaporation method, using ammonium bicarbonate as a porogen, followed by alkaline post-treatment to enhance the porosity. One gram of PLGA was dissolved in 20 mL of dichloromethane (DCM), with 20 μL of Span 80 as an emulsifier. The external aqueous phase was prepared by dissolving 1% (*w*/*v*) polyvinyl alcohol (70 kDa, partially hydrolyzed) in 100 mL of deionized water, with 200 μL of Tween 80. The inner aqueous phase consisted of 100 mg of ammonium bicarbonate dissolved in 2.5 mL of deionized water. This inner aqueous phase was added to the PLGA-DCM solution and emulsified by probe sonication to form the primary water-in-oil emulsion. This primary emulsion was immediately added dropwise to the external aqueous phase under magnetic stirring at 650 rpm for 10 min to form a water-in-oil-in-water (W/O/W) emulsion. The emulsion was stirred at 450 rpm for 8 h at room temperature for solvent evaporation and microsphere solidification. Then, the partially porous PLGA microspheres were collected and incubated in 50 mL of 0.2 N NaOH for 10 min to further leach the porogen and enhance porosity. A sieve was used to remove microspheres smaller than 75 μm. Microspheres were washed 3 times with PBS, then 3 times with deionized water.

*Polydopamine coating*. A total of 3 mL of porous PLGA microspheres were stirred overnight in 50 mL of dopamine solution (2 mg/mL in 10 mM Tris-HCl, pH 8.5) at 250 rpm and at 37 °C. Microspheres were washed 3 times in water. IL-2 was loaded onto polydopamine-coated PLGA microspheres, as described above for LC beads.

*PEI coating*. A total of 3 mL of porous PLGA microspheres were stirred overnight in 50 mL PEI solution (1 mg/mL in water) at 250 rpm and at 37 °C. Microspheres were washed 3 times in water. CpG-STAT3ASO^Cy3^ was loaded onto PEI-coated PLGA microspheres, as described above for LC beads.

*Scanning electron microscopy*. A few drops of PLGA microsphere solution were deposited on carbon conductive tabs, using a microcapillary tube, then dried at room temperature. The samples were then coated with gold and palladium (Au:Pd, 60:40 ratio) using a Cressington 308R coating system. The coated samples were imaged with a Sigma VP field emission scanning electron microscope (Zeiss, Oberkochen, Germany) at 5–15 keV.

### 2.5. Stabilizing Lipiodol Emulsions Using Emulsifiers

*Optimizing emulsion stability*. Initial emulsion optimization was performed using water-in-poppy oil emulsions. Various emulsifiers were dissolved in 4.5 mL poppy oil (lecithin, PEG-35 castor oil, Span 20, Tween 80, oleic acid, stearic acid). Various emulsifiers and additives were dissolved in 0.5 mL of water (NaCl, MgSO_4_, carboxymethyl–cellulose, Span 20, Tween 80, Tween 20) containing 0.5 mg/mL FD&C red 40. An emulsion was prepared using the pumping method with polypropylene syringes. The syringes were placed in a shaker at 37 °C and 80 rpm, and the separation half-life was determined by measuring the release of the aqueous phase.

*Stabilized drug-in-lipiodol emulsions*. The aqueous phase (0.4 mL) contained Visipaque 320 + 4 mg of CpG-STAT3ASO. The oil phase (3.6 mL) contained lipiodol (Guerbet, Villepinte, France) + 8% (*v*/*v*) Span 20 + 4% (*v*/*v*) Tween 80. A water-in-oil emulsion was formed using the pumping method (20 passes through a 3-way stopcock).

*Emulsion type and droplet size*. Emulsion type was evaluated by a water solubility test. A drop of emulsion was introduced into 1 mL of distilled water in a 1.5 mL Eppendorf tube at room temperature. If the emulsion dispersed into the water, this indicated an oil-in-water emulsion. If the emulsion remained in a separate phase, that indicated a water-in-oil emulsion. Droplet size was evaluated under light microscopy.

### 2.6. Transferring Drug-Loaded Microspheres into Lipiodol

Hydrated drug-loaded microspheres do not mix with lipiodol. To transfer drug-loaded microspheres into lipiodol, the water was replaced with acetone, which was then removed under vacuum prior to transfer to lipiodol.

Specifically, CpG-STAT3ASO-loaded quaternary amine microspheres were washed 3 times in acetone, then soaked in acetone overnight. The acetone supernatant was removed, and the microspheres were placed under vacuum for 2 to 3 h. Lipiodol was added, and residual acetone was removed under vacuum overnight.

### 2.7. In Vitro Drug Release Half-Life

Drug-loaded microspheres or lipiodol were eluted into PBS (pH 7.4) + 34.6% (*v*/*v*) glycerol (to match the viscosity of blood) at 37 °C and 80 rpm [23]. Eluted drug was measured at multiple time points (see Section 2.8). For elution from lipiodol, glass bottles were used to reduce background fluorescence. Time to elute 50% of the loaded drug was reported.

### 2.8. Drug Assays

CpG-STAT3ASO (Cy3-labeled) concentration was measured by fluorescence (excite 535 nm, emit 595 nm) or quantitative fluorescence microscopy (excite 550 nm, emit 605 nm). IL-2 and IFN-γ were measured by ELISA. Imiquimod was measured by absorbance at 245 nm, at pH 4.

### 2.9. Immunoembolization

The Institutional Animal Care and Use Committee at the Lundquist Institute approved all research procedures. All procedures and imaging were performed under general endotracheal anesthesia, using isoflurane. Euthanasia was performed by administering a barbiturate overdose intravenously.

Liver tumors (undifferentiated carcinomas) were induced in Oncopigs (Sus Clinicals, Chicago, IL, USA), as previously described [24,25]. Selective immunoembolization of the liver tumors was performed. Two liver tumors were embolized using the stabilized lipiodol/CpG-STAT3ASO emulsion. One liver tumor was embolized using CpG-STAT3ASO-loaded microspheres-in-lipiodol. Multiphase contrast-enhanced CT and necropsy were performed 1 week later, and tumors were harvested to evaluate the remaining drug concentration in the tumors (using quantitative fluorescence microscopy).

Liver tumor volume before and 1 week after treatment was measured. The change in liver tumor volume after immunoembolization (3 tumors in 1 pig) was compared to historical controls [24] that underwent bland embolization (6 tumors in 6 pigs), or no treatment (46 tumors in 17 pigs). Tumor growth rates were compared using *t*-tests, and *p* < 0.05 was considered statistically significant.

### 2.10. Burst Release

Burst release is the fraction of drug that is released systemically immediately after injection. Burst release of CpG-STAT3ASO was calculated from the plasma drug concentration, before and 5 min after immunoembolization, as previously described [4].

### 2.11. Safety

Safety was evaluated based on labs (CBC, comprehensive metabolic panel), imaging (multiphase contrast-enhanced CT of the abdomen and pelvis), daily clinical evaluation, and vital signs.

## 3. Results

### 3.1. Immunotherapy-Loaded Microspheres

**Figure 3:**
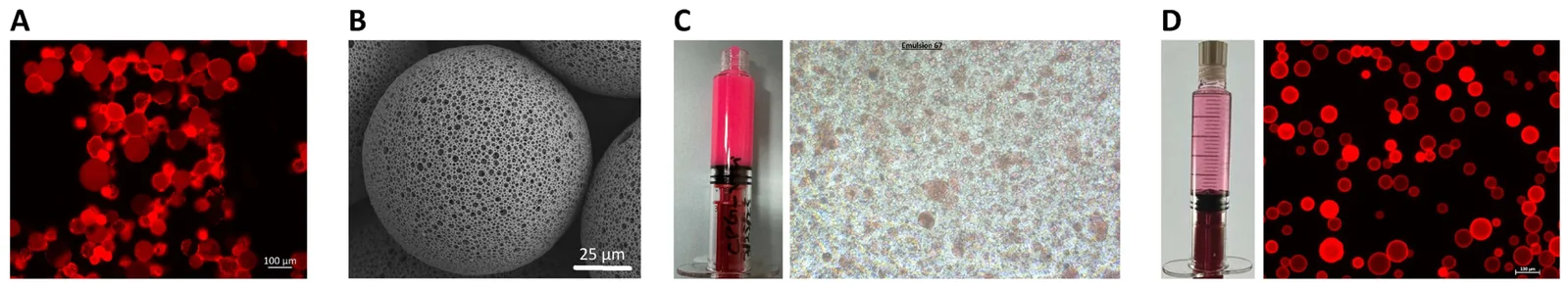
**Immunotherapy-loaded embolics**. (**A**). Fluorescence microscopy of CpG-STAT3ASO loaded onto protamine-coated LC beads. (**B**). Scanning electron microscopy of drug-loadable porous PLGA microspheres. (**C**). Photograph and photomicrograph of a drug-in-lipiodol emulsion, stabilized with Span 20 and Tween 80. (**D**). Photograph and fluorescence microscopy of CpG-STAT3ASO-loaded microspheres (45–165 µm) in lipiodol. The pink color of the microspheres is from a Cy3 label on the drug.

LC beads are negatively charged and can be loaded with positively charged immunotherapy agents (imiquimod), as well as cytokines (IL-2 and IFN-γ); see Table 1 and Table 2. Negatively charged immunotherapy agents—such as oligonucleotides or STING agonists—can also be loaded after coating LC beads with a polycation, such as protamine, in order to reverse the surface charge (Table 1, Figure 3A). For example, LC beads could only be loaded with 3.1 μg of CpG-STAT3ASO per mL of microspheres, but LC beads coated with protamine could be loaded with 5500 μg of CpG-STAT3ASO per mL of microspheres (Table 2).

Degradable PLGA microspheres (Figure 3B) can be coated with either polycations or polyanions, enabling loading of a wide range of different immunotherapy agents, including cytokines and oligonucleotides (Table 1 and Table 2).

### 3.2. Immunotherapy-Loaded Lipiodol

Another approach is to load immunotherapy agents into lipiodol. Lipiodol is attractive as an intra-arterial drug carrier [23,26], due to its ability to target multiple tumor types (HCC, colon cancer, lung cancer, pancreatic cancer, RCC, and others) in multiple organs (liver, lung, pancreas, kidney). To achieve extended release, lipiodol emulsions can be stabilized using emulsifiers, or drug-loaded microspheres can be suspended in lipiodol (Figure 3C,D).

Lipiodol emulsions were stabilized using various emulsifiers, and the emulsion type and stability were evaluated (Table 3). Without emulsifiers, lipiodol emulsions separated in 2.7 days, and with emulsifiers, lipiodol emulsions were stable for at least 2.8 weeks.

### 3.3. Drug Elution

**Figure 4:**
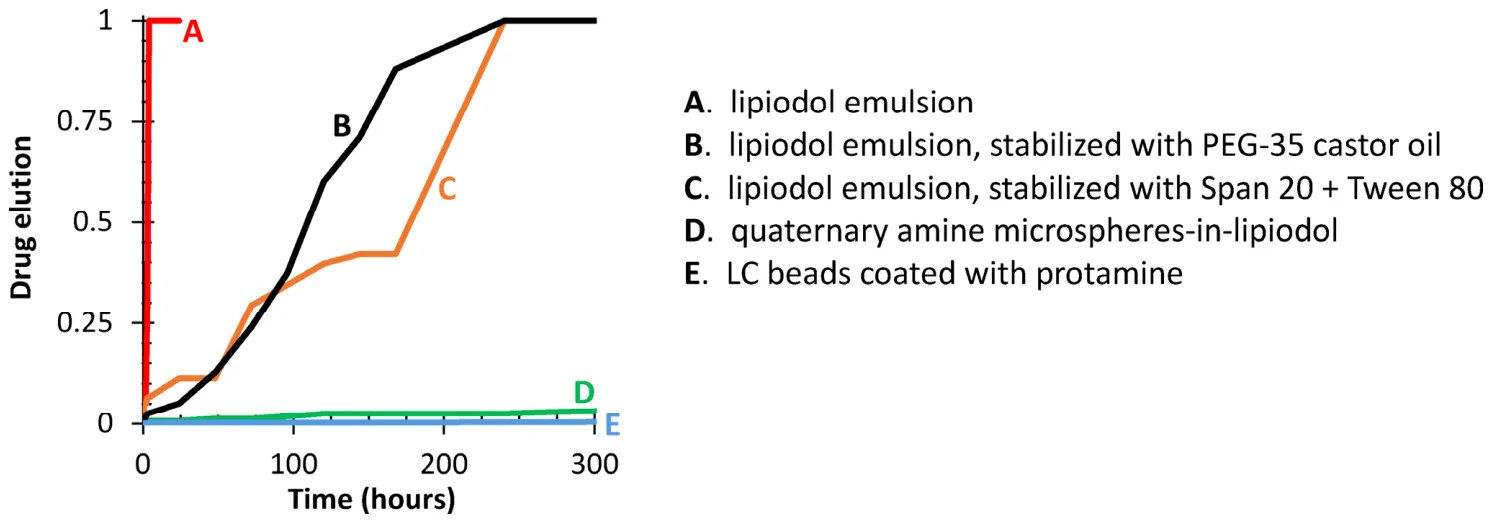
In vitro elution of CpG-STAT3ASO from various embolics. Drug is released from lipiodol emulsion in hours, from stabilized lipiodol emulsions in days, and from microspheres in weeks (Table 4).

In vitro, lipiodol emulsions without emulsifiers release drug in hours, stabilized lipiodol emulsions release drug in days to weeks, and drug-loaded microspheres release drug in >four weeks (Table 3 and Table 4, Figure 4). Thus, the desired drug release kinetics can be achieved by selecting the right embolic.

### 3.4. Immunoembolization of Liver Tumors

Based on drug release kinetics and translatability, two extended-release immunotherapy-eluting formulations were selected for further evaluation in a large animal model: stabilized lipiodol emulsions (using Span 20/Tween 80), and microspheres-in-lipiodol.

**Figure 5:**
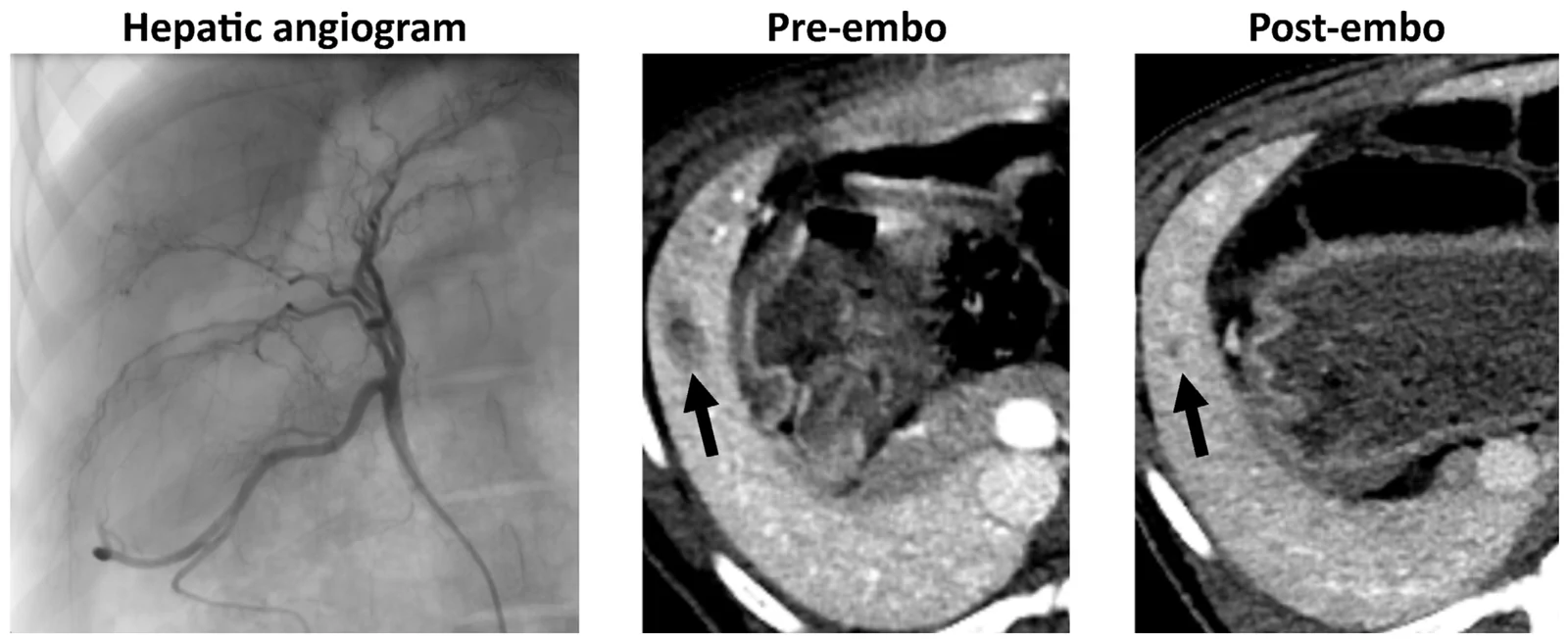
Immunoembolization of a pig liver tumor (arrows), using a lipiodol/CpG-STAT3ASO emulsion. CT scan one week after immunoembolization shows a radiographic response.

Immunoembolization was performed in an Oncopig (3.7-month-old female, 22 kg) with three liver tumors. Two of the tumors were embolized using 4 mL of lipiodol emulsion, containing 4 mg of CpG-STAT3ASO, stabilized with Span 20 and Tween 80 (Figure 5). All 4 mL were delivered, resulting in slow flow. One liver tumor was embolized using quaternary amine microspheres (45–165 μm), loaded with 2 mg of CpG-STAT3ASO, in 4 mL of lipiodol. Only 1 mL could be delivered before reaching stasis.

**Figure 6:**
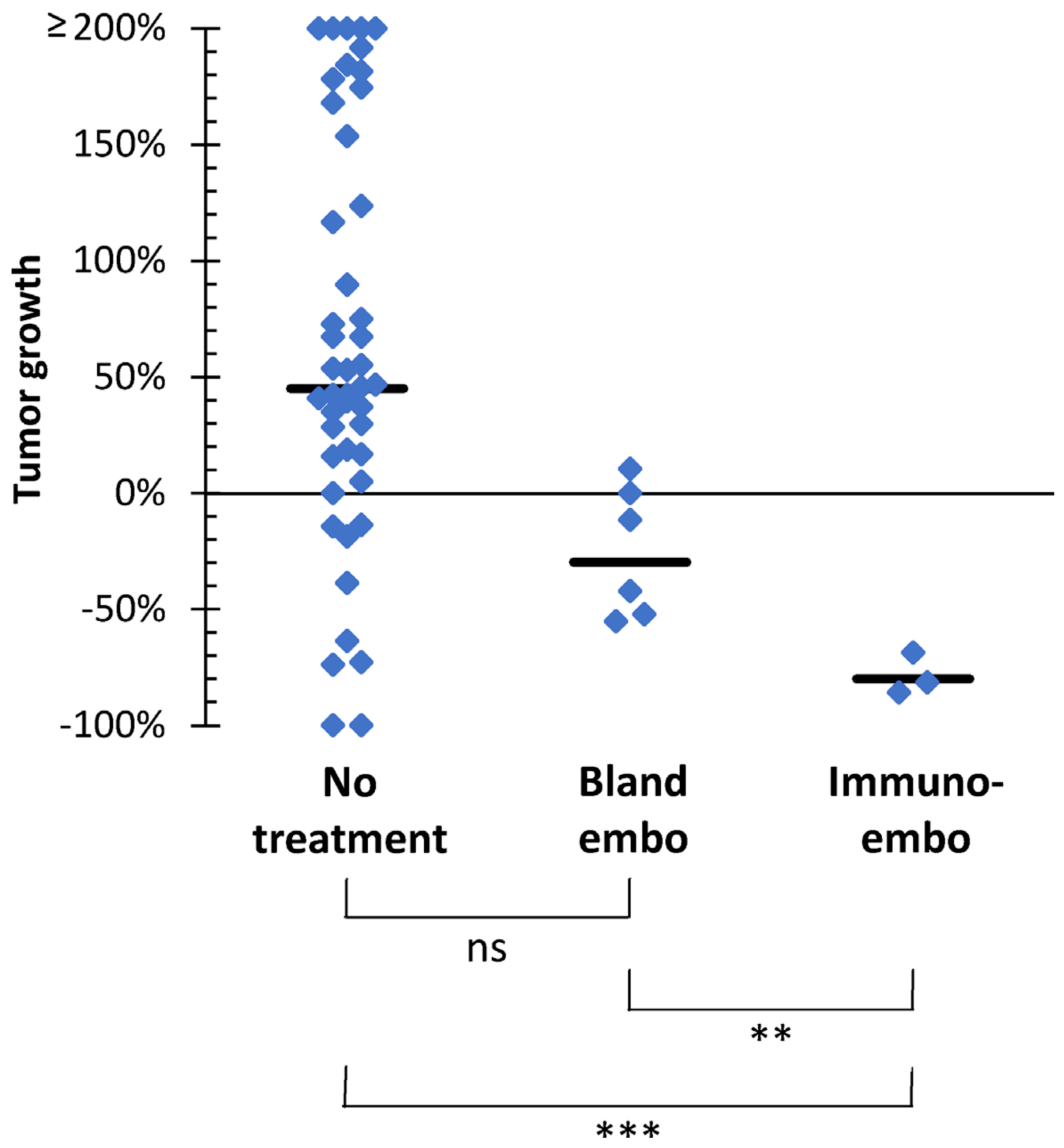
Change in liver tumor volume after immunoembolization compared with that historical controls that underwent on bland embolization, or no treatment [24]. Each point represents one liver tumor. ns (*p* > 0.05), * (*p* ≤ 0.05), ** (*p* ≤ 0.01), *** (*p* ≤ 0.001).

In this pilot study, liver tumors decreased in volume by 80% after immunoembolization, compared with a 30% decrease in volume after bland embolization (*p* = 0.003), and 45% growth in untreated liver tumors (*p* = 0.0009); see Figure 6. These encouraging results should be interpreted cautiously, given the small number of tumors treated.

### 3.5. Immunoembolization: Burst Release and Half-Life

After immunoembolization, burst release was measured based on the plasma concentration of CpG-STAT3ASO, five minutes after immunoembolization. Half-life was measured based on quantitative fluorescence microscopy to determine the CpG-STAT3ASO concentration in liver tumors, one week after immunoembolization.

The stabilized lipiodol emulsion resulted in 100% burst release in pigs, even though the emulsion was stable for >four weeks in vitro, with a 7.4-day drug release half-life in vitro (Table 4). Microspheres-in-lipiodol resulted in low burst release and a four-day half-life in pigs, compared to a> four-week half-life in vitro.

Thus, drug release in pigs is much faster than drug release in vitro. To simulate the high burst release seen with stabilized lipiodol emulsions, stabilized emulsions were injected through 10 μm polypropylene and nylon syringe filters to simulate passage through the capillary bed. Emulsions stabilized using Span 20/Tween 80 and PEG-35 castor oil both passed through the 10 μm filters without separating. We currently do not have any in vitro assays that can accurately predict in vivo burst release.

### 3.6. Immunoembolization: Safety

Based on clinical, laboratory, and imaging evaluation, there were no adverse events after immunoembolization. Specifically, there was no post-embolization syndrome, no immune-related adverse events, and liver function tests remained in the normal range after immunoembolization.

However, on necropsy, peritoneal adhesions were observed in association with immunoembolization using quaternary amine microspheres-in-lipiodol, but not with immunoembolization using the lipiodol emulsion.

## 4. Discussion

We present several methods for fabricating extended-release immunotherapy-loaded embolics: ion exchange microspheres, layer-by-layer microspheres, degradable PLGA microspheres, stabilized lipiodol emulsions, and microspheres-in-lipiodol. This diverse toolkit enables loading of a wide range of immunotherapy agents (cytokines, small molecules, and oligonucleotides), with release kinetics ranging from days to weeks in vitro, and up to four days in pig liver tumors. A pilot study of immunoembolization of liver tumors in a large animal model, using extended-release immunotherapy-loaded embolics, resulted in decreased tumor size, compared with bland embolization. Discrepancies between in vitro and in vivo drug release, and peritoneal adhesions seen with microspheres-in-lipiodol, highlight the importance of large animal studies for testing new immunotherapy-loaded embolics.

We used an iterative approach to design and validate these new immunotherapy-loaded embolics. First, we started with validated immunotherapy targets, with extensive rodent data demonstrating a benefit of intratumoral delivery [27,28,29,30]. Next, we designed hundreds of different immunotherapy-loaded embolic formulations, and evaluated and optimized these formulations based on technical criteria, such as drug loading, emulsion stability, microparticle clumping, and microcatheter injectability. Then, we evaluated in vitro drug elution for the top candidates. Finally, we tested the formulations in a pig model of liver cancer, evaluating safety, pharmacokinetics, and efficacy.

A previous study reported loading of positively charged immunotherapy agents onto DC Bead Lumi [31]. Here, we expand the range of agents that can be loaded onto DC beads or LC beads. We show that negatively charged immunotherapy agents (such as oligonucleotides or STING agonists) can also be loaded onto LC beads by first coating the microspheres with protamine to reverse the surface charge (layer-by-layer assembly).

Stabilized lipiodol emulsions have been studied extensively [5,32,33,34,35,36,37]. Here, we show that, despite emulsion stability for >four weeks and an in vitro drug release half-life of 7.4 days, the stabilized lipiodol emulsion released 100% of the immunotherapy agent within five minutes after immunoembolization of pig liver tumors. High burst release is also seen after chemoembolization in humans [23]. To address the burst release problem, we suspended dehydrated immunotherapy-loaded microspheres in lipiodol. Microspheres-in-lipiodol reduced burst release from 100% to <1%, with a half-life of four days in pig liver tumors.

To overcome the immunosuppressive tumor microenvironment, we performed transarterial immunoembolization of liver tumors, using extended-release lipiodol/CpG-STAT3ASO formulations. CpG-STAT3ASO is a first-in-class dual-function immunotherapy agent that both stimulates the anti-tumor immune response and counters immunosuppression in immunologically cold tumors [27,28,29,30]. The combination of TLR9 activation by CpG and STAT3 inhibition by the antisense oligonucleotide unleashes a potent immunostimulatory signal from within the tumor microenvironment that translates into systemic adaptive immune responses. Our new lipiodol/immunotherapy formulations release CpG-STAT3ASO into the tumor over four days, potentially generating sustained intratumoral immune activation (in situ vaccination). Local delivery maximizes immune activation in the tumor, while reducing systemic toxicity. Lipiodol is preferentially retained in liver tumors, and intra-arterial injection provides good coverage of large, heterogeneous, and multifocal tumors. In this pilot study in pigs, immunoembolization of liver tumors using extended-release lipiodol/CpG-STAT3ASO resulted in decreased tumor size, compared to bland embolization, with no autoimmune adverse events.

The immunotherapy-loaded embolics described here were designed for translatability to human trials. IL-2 and IFN-γ are FDA-approved for injection. Imiquimod is FDA-approved for topical use. Lipiodol is FDA-approved for use in the hepatic artery. LC beads are FDA-cleared for embolization of hypervascular tumors. PLGA, protamine, Span 20, Tween 80, and PEG-35 castor oil are FDA-approved excipients for injection.

This study has several limitations. First, the in vivo component was an exploratory pilot study involving a single animal with three treated tumors, and outcomes were compared against historical controls. The animal results should be considered hypothesis-generating. Second, follow-up was limited to one week, so delayed toxicity was not evaluated. Third, substantial differences between in vitro and in vivo release kinetics indicate that the current in vitro drug release models are inadequate.

## 5. Conclusions

We developed methods for loading a wide range of immunotherapy agents (CpG-STAT3ASO, imiquimod, IFN-γ, and IL-2) onto LC beads, lipiodol, and PLGA microspheres. The loading method must be tailored to the drug, the embolic, and the desired release kinetics. Immunoembolization of liver tumors using extended-release lipiodol/CpG-STAT3ASO demonstrated promising preliminary results in a large animal model, supporting further pre-clinical studies of this new strategy for cancer immunotherapy.

## Figures and Tables

**Table 1:** **Zeta potential of microspheres**. LC bead charge can be reversed by coating with protamine, enabling loading of negatively charged drugs. Note that LC beads get smaller when coated with protamine. PLGA microsphere charge can be reversed by coating with PEI, enabling loading of negatively charged drugs. See Table 2.

Microsphere	Coating	Zeta Potential (mV)	Size (μm)
Quaternary amine (+)	(none)	59.9	45–165
LC beads (−)	(none)	−13.5	131 ± 52
	protamine (+)	24.4	82 ± 29
Porous PLGA	(none)	−17.6	80–110
	polydopamine (−)	−27.5	80–110
	polyethylenimine (+)	33.8	80–110

**Table 2:** **Immunotherapy-loaded microspheres**. Positively charged drugs, such as imiquimod, can be directly loaded onto LC beads. Negatively charged drugs, such as CpG, can be loaded onto LC beads by first coating the beads using a positively charged polymer, such as protamine. PLGA microspheres can be coated with either polyethyleneimine or polydopamine, then loaded with either negatively or positively charged drugs. Drug loading onto quaternary amine microspheres (previously reported [4]) is shown for comparison.

Drug	Microsphere	Microsphere Coating	Drug Loading (μg Drug/ mL Microspheres)	% Loading
CpG-STAT3ASO (−)	LC beads (−)	(none)	3.1	14%
	LC beads (−)	protamine (+)	5500	99%
	Porous PLGA	polyethyleneimine (+)	2000	79%
	Quaternary amine (+)	(none)	4100	99%
3′,3′-cGAMP (−)	Quaternary amine (+)	(none)	3800	92%
Imiquimod (+)	LC beads (−)	(none)	2000	80%
IL-2 (±)	LC beads (−)	(none)	0.95	95%
	Porous PLGA	polydopamine (−)	1.0	100%
	Quaternary amine (+)	(none)	0.98	98%
IFN-γ (±)	LC beads (−)	(none)	79	98%
	Porous PLGA	polyethyleneimine (+)	22	99%
	Quaternary amine (+)	(none)	16	95%

**Table 3:** **Stabilized lipiodol emulsions release CpG-STAT3ASO over days to weeks in vitro**. One hundred and one different formulations were tested, and the top candidates were selected based on drug release kinetics, translatability, emulsion type, and injectability.

Emulsifier	Emulsion Type	Droplet Size (µm)	Emulsion Separation Half-Life	Drug Release Half-Life
none	o/w/o	317	2.7 days	3.3 h
PEG-60 hydrogenated castor oil	w/o	<1	2.8 weeks	>4 weeks
Span 80 + Tween 80 *	w/o	35.6	>4 weeks	31 h
hydrogenated PC + Tween 80 *	w/o	4.6	>4 weeks	30 h
PEG-35 castor oil *	w/o	1–5	>4 weeks	4.5 days
Span 20 * + Tween 80 *	w/o	20–25	>4 weeks	7.4 days
quaternary amine microsphere	microsphere/oil	45–165	>4 weeks	>4 weeks

* FDA approved for injection, and USP/NF grade commercially available; PC = phosphatidylcholine.

**Table 4:** In vitro versus in vivo (immunoembolization of pig liver tumors) release of CpG-STAT3ASO from various embolics.

Embolic	In Vitro		In Pig Liver Tumors	
	Burst Release	Half-Life	Burst Release	Half-Life
A. Lipiodol emulsion	1.0%	3.3 h		
B. Lipiodol emulsion, stabilized with PEG-35 castor oil	0.68%	4.5 days		
C. Lipiodol emulsion, stabilized with Span 20/Tween 80	4.1%	7.4 days	100%	n/a
D. Quaternary amine microspheres-in-lipiodol	0.15%	>4 weeks	0.83%	4.0 days
E. LC beads coated with protamine	0.07%	>4 weeks		

## Data Availability

The raw data supporting the conclusions of this article will be made available by the authors on request.
